# Factors Affecting Voluntary Self-Isolation Behavior to Cope with a Pandemic: Empirical Evidence from Colombia vs. Spain in Times of COVID-19

**DOI:** 10.3390/bs11030035

**Published:** 2021-03-15

**Authors:** Diana Escandon-Barbosa, Andrea Hurtado, Alina Gomez

**Affiliations:** 1 Departamento Gestion de Organizaciones, Pontificia Universidad Javeriana Cali, 760030 Cali, Colombia; 2 Universidad “Antonio Jose Camacho”, 760007 Valle del Cauca, Colombia; anday490@yahoo.com; 3 Departmento Contabilidad y Finanzas, Pontificia Universidad Javeriana, 111611 Bogotá, Colombia; aligomez@javerianacali.edu.co

**Keywords:** self-isolation, COVID-19, multi-group, volitional behavior

## Abstract

Global pandemics are not a new phenomenon. They have occurred at different points in time and can be of different scales. COVID-19 appeared in 2020 and its spread has reached more than 60 countries worldwide. This research aims to analyze voluntary self-isolation behavior used to cope with the COVID-19 pandemic. As part of this study, we carried out sampling in Colombia and Spain, which share similar cultural characteristics but which have substantial social and economic differences. A multi-group model was used to test the application of the theory of planned behavior and the theory of reasoned action in order to analyze self-isolation behavior. The results show that there are differences in self-isolation behavior between both countries, especially with regard to attitudes towards self-isolation and volitional behavior.

## 1. Introduction

COVID-19 appeared in Wuhan, China at the beginning of December 2019 and quickly spread to other parts of China and neighboring countries and finally reached different countries around the world. Due to its rapid growth in number of cases, the World Health Organization (WHO) declared COVID-19 an outbreak on 11 March 2020. From the daily situation reports of the WHO, it becomes evident how COVID-19 evolved into a global crisis in every respect and how WHO’s public health strategic objectives changed from simple to intricate measures. COVID-19 has led local and national authorities around the world to try to curb its spread by implementing a series of measures that restrict daily life, and by carrying out awareness campaigns to encourage people to adopt voluntary isolation [1]. According to Ajzen and Fishbein (2005) [2], all the behavioral, normative, and control beliefs that individuals possess and affect the adoption of a given behavior, are influenced by other external cultural, personal, and social factors. These include gender, education, age, economic status, physical environment, access to information, values, prejudices, and individual and collective orientations. For the purposes of this article (in which the behavior to be predicted is the will to self-isolate repetition), we used the study by Zhang et al. (2020) [1] as a basis in which such behavior was seen to be affected by other factors, i.e., gender differences, age, education, personality traits, economic status, and marital status. This paper seeks to investigate the presence of different patterns of behavior according to the conditions in which the virus has occurred in different regions.

With this aim in mind, a model was proposed to explain self-isolation behavior by comparing the cases of Colombia and Spain, taking into account that in most European countries the infection curve had a slower start than in Latin American countries [3]. After the virus hit the Asian continent, Europe became the epicenter of the outbreak. In mid-April, Italy and Spain were the most affected countries on the continent with mortality rates above 10% and more than 200,000 positive cases. By the same date, Colombia was close to 4000 positive cases and a mortality rate of 4.75%. Both Spain and Colombia had already adopted their respective isolation measures by that date.

## 2. Conceptual Model and Hypotheses

Various theories explain social behavior, all of which are based on the study of intentions as predictors of such behavior [4]. Among these theories are social cognitive theory, the information–motivation–behavioral skills model [5], interpersonal relations theory, and subjective culture [6]. However, much of the research on social behavior explained through intentions has been carried out in the context of the theory of reasoned action [7,8,9] and the theory of planned behavior [10,11]. The theory of reasoned action was initially presented by Fishbein and Ajzen (1975) [7] and subsequently developed and tested by the same authors [8]. It is a general theory of human behavior that relates attitudes, beliefs, and behavior that are related when making decisions at the behavioral level. According to this theory, individuals are rational by nature, which allows them to use the available information to carry out their behaviors. The authors suggest that intention is the best predictor of behavior, but it is also important to take into account skills and abilities, as well as environmental factors (behavior control). According to this theory, people develop a behavior because they have the required skills and abilities and there are no environmental forces to prevent them from carrying out these intentions. In other words, they are in control of the current situation. In the model initially proposed by Fishbein (1967) [9], intention is a function of the subjective norms and attitudes towards behavior. This is because individuals attempt to carry out a behavior when they evaluate that it is positive and believe that other subjects should also carry it out [7,8]. On the other hand, attitude towards behavior is a function of the behavioral beliefs of individuals, while the subjective norms are a function of the normative beliefs. These are born from the influence that norms have for the realization of the actions of an individual.

The theory of planned behavior is an extension of the theory of reasoned action [9,12]. It is a cognitive theory that allows individuals to predict and identify behavioral intentions with a high degree of accuracy. This theory proposes that an individual is influenced by three factors: attitudes towards behavior, subjective norms regarding behavior, and perceived control over behavior. This theory has usually been used to predict health-related behaviors such as work, sexual, and eating behaviors [13,14,15]. Therefore, this theory is appropriate for studying individuals’ intentions in the face of pandemic risk.

**Figure 1:**
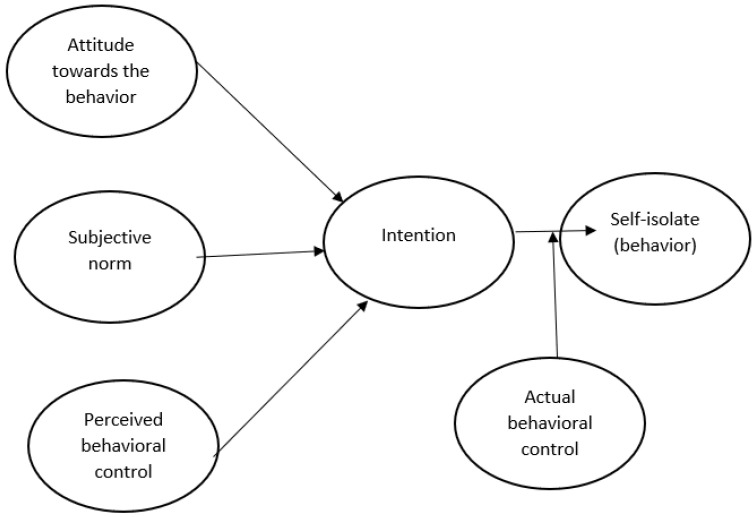
Conceptual model.

In this study, the model in Figure 1 (proposed by Ajzen and Fishbein (2005) [2]) is based on the causal structure that exists between the theory of reasoned action and the theory of planned behavior. In this model, the antecedents of intentions and behavior are represented. It illustrates intention as the antecedent of real behavior, which is in turn is determined by attitudes towards behavior, subjective norms, and control of perceived behavior. On the other hand, control of actual behavior (or volitional control) is expected to moderate the relationship between intention and behavior in such a way that the greater the volitional control, the stronger the effect of intention on behavior. The behavior to be predicted for the purposes of this article corresponds to the desire for self-isolation.

In this model, attitude towards behavior is the degree to which an individual has a favorable or unfavorable assessment of self-isolating behavior [11]. Subjective norms refer to an individual’s perception of social pressure to adopt a certain behavior [11,16]. Control of perceived behavior is the perceived ease or difficulty of the individual to adopt a behavior [11]. On the other hand, control of current behavior or volitional control refers to an individual’s ability to self-control in the face of a person’s own acts [9]. Finally, an individual’s intention is the central factor in adopting a certain behavior, so the greater the intention, the greater the probability that the behavior will be adopted [11].

Therefore, through the variables presented in Figure 1 that make up the proposed model (in accordance with the theory of reasoned action and the theory of planned behavior), this paper seeks to understand an individual’s intention to voluntarily adopt self-isolating behavior as a result of the current pandemic caused by COVID-19.

Several studies have sought to test this model empirically. Zhang et al. (2020), Zhang and Wang (2015), and Armitage and Conner (2001) [1,13,17] found that intentions can be predicted fairly accurately from attitudes to behavior, subjective norms and perceived behavioral control. There is also evidence that individuals consistently differ in the importance that they give to attitudes towards behavior, subjective norms, and perceived behavioral control [18]. For their part, Cook et al. (2018), Rashid et al. (2015), and Flahault et al. (2006) [19,20,21] studied the impact of self-isolation in preventing or delaying the spread of a pandemic. In view of these studies, the following hypotheses are proposed:

**Hypothesis** **1** **(H1).** *Attitudes towards behavior positively influence the intention to voluntary self-isolate*.

**Hypothesis** **2** **(H2).** *Subjective norms positively influence the intention to voluntary self-isolate*.

**Hypothesis** **3** **(H3).** *Perceived behavioral control positively influences the intent of voluntary self-isolation*.

Similarly, intentions are usually good predictors of behavior under the influence of volitional control [18]. That is to say, the greater an individual’s ability to control his or her actions, the greater the influence of intentions are on behavior [2]:

**Hypothesis** **4** **(H4).** *Volitional control moderates the relationship between intentions and voluntary self-isolating behavior*.

Older individuals are more likely to adhere to preventive measures [22]. However, less educated individuals are less likely to understand the importance of adhering to social norms in a pandemic [23]. For Roger et al. (2015) [24], the socioeconomic level of an individual is also a determinant of behavior during a pandemic. Another factor is living with family members who require special attention such as children, the elderly, the disabled, or people with chronic diseases, as this leads to a perceived increased risk of infection [25].

## 3. Methodology

The unit of analysis used in this research are residents over 18 years in Colombia and Spain, between March and May 2020. As part of the conditions for participation, a telephone survey was carried out in both countries. A company that carries out surveys randomly selected phone numbers to achieve a sample of 190 participants in Spain and 250 in Colombia (see Table 1).

The selected sample is representative of the population parameters for gender, age, and educational level for the countries participating in the sample. Of the 190 people spoken to in Spain, 51.05% were women and 48.9% men. More than 70% had or were studying in the higher levels of education and were over 40 years of age. These data are consistent with population information from the National Institute of Statistics (INE). The sample of Colombians taken for this study is also in line with population figures from the Colombian National Department of Statistics (DANE); 53% were women and 47% men and more than 45% were in the 25–54 age group. Respondents’ educational levels were; 58.8% undergraduate, 31.2% master’s degree level and 10% doctorate level.

The choice of Colombia and Spain is associated with the need to make comparative analyzes between objects of study, which allowed aspects of diversity and similarity to be brought together. In this study, diversity was achieved because the pandemic began at different points in time. In Spain, it began more than three weeks earlier than in Colombia. This situation may have influenced the information and decision-making process surrounding the COVID-19 pandemic. Another difference between the two countries is the economic conditions of the inhabitants, which may influence their decision to isolate themselves for a longer period of time.

Regarding similarity, Colombia and Spain are culturally similar countries, creating a group of respondents who can assimilate the effect of a pandemic with similar behaviors or beliefs. Additionally, Colombia and Spain are statistically different and showed adequate level to understand this topic. The F-Value was 6430 and it was significantly different from 1. Thus, we rejected H0 of average equality.

The project developed in 2020, “Factors affecting voluntary self-isolation behavior to cope with a pandemic: empirical evidence from Colombia vs. Spain in times of COVID-19”, received the endorsement of the Research and Ethics Committee of the Faculty of Economic Sciences and Administrative, of the Pontifical Universidad Javeriana Cali, which was developed according to the Helsinki declaration and approved consecutively (DECA-88-2021).

For data analysis, the structural equation system (SEM) and Lisrel Software version 9.3 were used. A multi-group model was made with 6 constructs: subjective norms (SN), control of perceived behavior (CPB), intention (I), attitude through behavior (ATB), control of volitional behavior (CVB), and behavior in self-isolation (BS). This multi-group showed values for Colombia and Spain.

The calculation of moderating effects must be calculated from the combination between the items that make up each latent variable. Therefore, according to Ping (1996), for latent variables *X* and *Z* with indicators *x*_1_, *x*_2_, *z*_1_, and *z*_2_ a new latent variable *XZ* must be specified with the combination of the items of the original latent variables as follows: *x*_1_*z*_1_, x_1_*z*_2_, *x*_2_*z*_1_, and *x*_2_*z*_2_. In the case of this research that tries to explain the moderation of CVB (Control of volitional behavior) to I and BS, we combined items of the two latent variables. In general, the model of quadratic effects under the structural equation methodology can be visualized in the following way.

$$\begin{array}{c} V\alpha r(x_{1}z_{1})\;=\;V\alpha r[(\lambda_{x1}X\;+\;\epsilon_{x1})(\lambda_{z1}Z\;+\;\epsilon_{z1})]\\ =\;\lambda_{x1}^{2}\;\lambda_{z1}^{2}\;V\alpha r(XZ)\;+\;\lambda_{x1}^{2}V\alpha r(X)V\alpha r(\epsilon_{z1})]\\ +\;\lambda_{z1}^{2}\;V\alpha r(Z)V\alpha r(\epsilon_{x1})\;+\;V\alpha r(\epsilon_{x1}V\alpha r(\epsilon_{z1})\\ =\;\lambda_{x1}^{2}\lambda_{z1}^{2}[V\alpha r(X)V\alpha r(Z)\;+\;{Cov}_{2}(X,Z)]\\ +\;\lambda_{z1}^{2}V\alpha r(X)V\alpha r(\epsilon_{z1})\;+\;\lambda_{z1}^{2}V\alpha r(Z)V\alpha r(\epsilon_{z1})\\ \;+\;V\alpha r(\epsilon_{x1})V\alpha r(\epsilon_{x1})\\ V\alpha r(x_{1}x_{1})\;=\;V\alpha r[(\lambda_{x1}X\;+\;\epsilon_{x1})(\lambda_{x1}X\;+\;\epsilon_{x1})]\\ =\;\lambda_{x1}^{2}\;\lambda_{x1}^{2}\;V\alpha r(X^{2})\;+\;4\lambda_{x1}^{2}V\alpha r(X)V\alpha r(\epsilon_{x1})\\ +\;V\alpha r(\epsilon_{x1})\\ +\;2{V\alpha r}^{2}(\epsilon_{x1}) \end{array}$$

## 4. Measures

This study used three scales of the reflective type. These scales, according to Diamantopoulos (1999) [26], are the most often used in the social sciences and when the observed variables are manifestations of constructs. Therefore, the scales used were: subjective norms (SN), control of perceived behavior (CPB), intention (I), attitude through behavior (ATB), control of volitional behavior (CVB), and behavior in self-isolation (BS).

As previously mentioned, this study used as its basis the constructs raised by the theory of planned behavior to determine its applicability to the COVID-19 context in Colombia and Spain. The attitude scale contained nine items in which respondents were asked about the conditions of the COVID-19 pandemic situation and the way in which they perceive it.

The subjective norms scale has four items in which the norms internalized in society were evaluated [7,8]. This scale showed a good reliability and validity (alpha Cronbach: 0.89); it was used in different papers related with psychology topics when it was necessary know about people who internalized social norms [11,16].

Control of perceived behavior was inspired by Ajzen, 1991 [11], who used nine questions that examine how the respondent could modify or lean towards a behavior associated with a particular situation. Our data showed that control of perceived behavior adjusted well (alpha Cronbach: 0.91).

The intention scale was measured using four questions that evaluated the behavioral options that a subject might experience under certain circumstances [11]. This scale obtained good reliability (alpha Cronbach: 0.88).

In the case of the behavior scale, we associated it with the existence of self-isolation, as an option for inhabitants in Colombia and Spain, specifically in the context of the pandemic [19,20,21]. Its alpha Cronbach was 0.92.

Lastly, the scale of volitional control of behavior was added as a moderating variable, which seeks to measure the impulsive behavior of an individual in a specific situation [2,27].

All scales were measured on a scale of 1 to 7, for which 1 was “totally disagree” and 7 was “totally agree”. Factor analysis yielded a reasonable fit of data for both the general model and the multi-group model, because all measurements showed adequate reliability. The score compose reliability (SCR) was greater than 0.6 and the average variance extracted (AVE) was greater than 0.5. The control of perceived behavior construct showed the highest values for both SCR and AVE, as opposed to the intention construct, which reported values close to the established minimum limits (See Table 2).

Moreover, all the loads corresponded to their hypothetical factors, and the estimates were very significant, since the T-value presented high values that provided evidence of convergent validity. In this sense, it can be seen that the construct of subjective norms and control of perceived behavior had the highest factorial faults. This gave a higher level of validity to the use of this construct and its respective items. On the other hand, discriminant validity was confirmed for all scales since it was possible to verify that the value “1” is not found in the confidence intervals calculated between each pair of constructs. In addition, discriminant validity was also confirmed because, in each scale, the average of the variance extracted by the underlying construct was greater than the variance shared with another latent construct.

This study used different mechanisms to mitigate the bias of the common method. First, the items in the instrument were reversed and the order of the questions in the questionnaire was counterbalanced.

## 5. Results

Initially, a comparison was established between the multi-group model of the structural equations and the model for the total sample without the groups. After observing the indicators, we concluded that the adjustments of the two models were satisfactory. In both cases, the RMSEA (Residual Measure) was below the 0.08 threshold and the measure of incremental fit construct (CIF) was above 0.9. However, the comparison between the two models allowed us to identify the multi-group model with the highest quantitative and theoretical quality (see Table 3).

Firstly, the adjustment of the multi-group model, which had more degrees of freedom, was significantly better in terms of chi-square (chi-square difference = 423,804, for 105 degrees of freedom, *p* < 0.001). Additionally, the rest of the adjustment indicators considered did not achieve significant differences between the two models. In this sense, the adjustment indicators were adequate in both models. The multi-group model then became the basis for an explanation of the proposed hypotheses, and the existence of globally significant differences for the analysis in Colombia and Spain were accepted.

Hypothesis 1 was confirmed, where it was stated that the attitude towards behavior positively influences the intention for voluntary self-isolation for the Colombian sample (β_32_ = 0.26; *p* < 0.01) and the Spanish sample (β_32_ = 0.49; *p* < 0.01). With respect to Hypothesis 2, it was established that there was only a direct relationship between the subjective norms and the intention for voluntary self-isolation in Spain (β_13_ = 0.52; *p* < 0.01). However, that was not confirmed in the Colombia sample (β_13_ = 0.19; *p* > 0.1). In this sense, it was possible to provide evidence for the existence of significant differences in the results of this relationship with respect to the two samples (*t* = 2.54; *p* < 0.01). Additionally, Hypothesis 3 was verified, where it was stated that perceived behavioral control has a positive influence on intention for voluntary self-isolation, both for Colombia (β_13_ = 0.27; *p* < 0.1) and Spain (β_13_ = 0.26; *p* < 0.1).

Hypothesis 4 affirmed that there was a moderating relationship between volitional control in the relationship between intention and behavior for voluntary self-isolation for Colombians (β_22_ = 0.32; *p* < 0.1) and Spaniards (β_22_ = 0.52; *p* < 0.1). It was possible to detect significant differences in this relationship for the two different sectors (*t* = 10.05; *p*< 0.01).

## 6. Discussion

With regards to the willingness to self-isolate in the context of a pandemic, it should be mentioned that isolation and the development of quarantines have been developed by different countries. However, awareness and radical compliance with these measures can also be affected by volitional behavior. This study demonstrates that a greater attitude towards self-isolation behavior was achieved in Spain. This difference with the sample in Colombia proves what Ajzen (1991) stated, since the experiences in Spain in terms of the number of infected people could influence the intention towards self-isolation behavior. In the case of Colombia, the figures have been much lower and the peak of the pandemic was not reached by May. 

In the case of subjective norms, we can conclude that in Spain and in Colombia they did not manage to influence the intention of self-isolation. This finding is relevant because evidence was generated regarding the low social pressure to develop this behavior in both countries. The role of family and friends may not have been conclusive or determinant in making the decision for this behavior. This proves that many of people’s behaviors are self-reflective and that they do not necessarily consult those who are closest in their social circles.

Therefore, in this study it was possible to prove one of the postulates of the planned behavior theory, in which it is showed that individuals assign different levels of relevance to attitudes towards behavior, the subjective norms, and the perceived behavioral control. In this sense, relevance depends, as Finlay et al. stated (1999) [18], on the type of behavior that one seeks to predict. In the case of self-isolation in the midst of a pandemic, attitude and volitional control become the most relevant aspects at the time of decision-making.

Concerning the limitations to this study, the model used was a cross-sectional type and therefore explain long-term effects of different constructions. In addition, the authors recognize the scope of data collection as limited, because sampling was done via telephone in Spain and Colombia. Therefore, the registered sample size is recognized as another limitation. Nonetheless, owing to the size of the model, the achievement of the groups from different countries and the objectives of the research are relevant.

Regarding future lines of research, it would be beneficial to carry out this research in other countries in order to make more general analyzes or conclusions about the study variables. In addition, the way in which the data for this study was gathered prevents us from capturing the effect of educational level or other social variables that may be relevant in future research, and which are likely to impact on the intention to carry out self-isolation behavior.

## 7. Conclusions

This article aimed to analyze the factors involved in the decision to self-isolate, especially those factors that encourage people to voluntarily choose this option. There was little evidence about the self-isolation processes associated with pandemic issues or the extreme need for health care. However, this study follows the line of Mas et al. (2012) [28] who proved that the decision to self-isolate was not only made based on government regulation but also depended on interpersonal factors such as self-care or fear. Another relevant finding was that the decision to self-isolate was greater in Spain than in Colombia, which could be attributed to the existence of social conditions that allowed this decision to be taken to a greater extent in countries with higher income levels. This finding allows us to verify what Eastwood et al. (2010) [29] proposed.

Therefore, in this study we verified the influence of the country on the results obtained from the theory of planned behavior in a pandemic context, as well as the possibility of assuming self-isolation as a protective behavior. This multi-group structure sheds light on differences that exist at the time of applying the theory of planned behavior in two culturally similar countries, but which differ vis-à-vis quality of life.

As a clarification, and to ensure a correct understanding of the results, there was an invariance in the measurement parameters for the constructions used in this article. Therefore, these concepts were perceived in the same or almost identical way for Colombians and Spaniards.

On the other hand, we highlighted the relationship of moderation of volitional control in the relationship between intentions and voluntary self-isolating behavior. This type of finding is relevant because in both Colombia and in Spain it was found that people who declare that they have greater self-control in their impulses may have a greater intention to exhibit voluntary self-isolation behavior. Therefore, they may change their usual behavior (continuing with their normal routine) and increase their intention to later perform voluntary self-isolation. This relationship is stronger in Spain and is associated with an individual’s ability for greater self-control in relation to their actions, in accordance with Ajzen and Fishbein (2005) [2].

## Figures and Tables

**Table 1:** Characteristics of participants.

	Colombia	Spain
Number of participants	250	190
Age	Over 18 years	Over 18 years
Women	53%	51.05%
Men	47%	48.9%
Educational levels	58.8% UG 31.2% MDL 10% DL	70% HLE

UG: undergraduate; MDL: master’s degree level; DL: doctorate level; HLE: higher levels of education.

**Table 2:** Measurement model.

	General Model			Colombians			Spaniards		
Constructs	Estimate	SCR	AVE	Estimates	SCR	AVE	Estimates	SCR	AVE
I	0.81	0.91	0.8	0.91	0.92	0.72	0.94	0.97	0.81
SN	0.83	0.92	0.82	0.89	0.93	0.81	0.87	0.92	0.80
CPB	0.60	0.68	0.53	0.57	0.74	0.61	0.55	0.73	0.60
CVB	0.81	0.90	0.81	0.87	0.90	0.77	0.86	0.90	0.76
ATB	0.93	0.95	0.80	0.93	0.79	0.69	0.83	0.91	0.78
BS	0.84	0.90	0.79	0.90	0.77	0.68	0.84	0.90	0.76

SCR: score compose reliability; AVE: average variance extracted; I: intention; SN: subjective norms; CPB: control of perceived behavior; CVB: control of volitional behavior; ATB: attitude through behavior; BS: behavior in self-isolation.

**Table 3:** Structural model.

Relationship with the Model	Colombians			Spaniards	
ATB →I	β_31_	0.75	9.04 ***	0.59	4.69 ***
SN→I	β_13_	0.19	1.86	0.52	5.10 ***
CPB→I	β_21_	0.27	4.70 ***	0.26	2.70 ***
I→BS	β_32_	0.26	3.54 ***	0.49	7.74 ***
CVB→BS	β_42_	0.28	3.78 ***	0.43	6.56 ***
I*CVB→BS	β_52_	0.32	4.33 ***	0.52	6.76 ***
	X^2^ = 745.84				
	CIF = 0.953				
	RMSEA = 0.0348				

*** *p* < 0.01. I: intention; SN: subjective norms; CPB: control of perceived behavior; CVB: control of volitional behavior; ATB: attitude through behavior; BS: behavior in self-isolation; CIF: incremental fit construct; RMSEA: root mean square error of approximation.
